# Tau-related grey matter network breakdown across the Alzheimer’s disease continuum

**DOI:** 10.1186/s13195-021-00876-7

**Published:** 2021-08-13

**Authors:** Wiesje Pelkmans, Rik Ossenkoppele, Ellen Dicks, Olof Strandberg, Frederik Barkhof, Betty M. Tijms, Joana B. Pereira, Oskar Hansson

**Affiliations:** 1 Alzheimer Center Amsterdam, Department of Neurology, Amsterdam Neuroscience, Vrije Universiteit Amsterdam, Amsterdam UMC, Amsterdam, The Netherlands; 2 Clinical Memory Research Unit, Department of Clinical Sciences, Lund University, Malmö, Sweden; 3 Department of Radiology & Nuclear Medicine, Amsterdam Neuroscience, Vrije Universiteit Amsterdam, Amsterdam UMC, Amsterdam, The Netherlands; 4 Queen Square Institute of Neurology and Centre for Medical Image Computing, University College London, London, UK; 5 Division of Clinical Geriatrics, Department of Neurobiology, Care Sciences and Society, Karolinska Institute, Stockholm, Sweden; 6 Memory Clinic, Skåne University Hospital, Malmö, Sweden

**Keywords:** Alzheimer’s disease, Graph theory, Grey matter network, MRI, PET, Tau

## Abstract

**Background:**

Changes in grey matter covariance networks have been reported in preclinical and clinical stages of Alzheimer’s disease (AD) and have been associated with amyloid-β (Aβ) deposition and cognitive decline. However, the role of tau pathology on grey matter networks remains unclear. Based on previously reported associations between tau pathology, synaptic density and brain structural measures, tau-related connectivity changes across different stages of AD might be expected. We aimed to assess the relationship between tau aggregation and grey matter network alterations across the AD continuum.

**Methods:**

We included 533 individuals (178 Aβ-negative cognitively unimpaired (CU) subjects, 105 Aβ-positive CU subjects, 122 Aβ-positive patients with mild cognitive impairment, and 128 patients with AD dementia) from the BioFINDER-2 study. Single-subject grey matter networks were extracted from T1-weighted images and graph theory properties including degree, clustering coefficient, path length, and small world topology were calculated. Associations between tau positron emission tomography (PET) values and global and regional network measures were examined using linear regression models adjusted for age, sex, and total intracranial volume. Finally, we tested whether the association of tau pathology with cognitive performance was mediated by grey matter network disruptions.

**Results:**

Across the whole sample, we found that higher tau load in the temporal meta-ROI was associated with significant changes in degree, clustering, path length, and small world values (all *p* < 0.001), indicative of a less optimal network organisation. Already in CU Aβ-positive individuals associations between tau burden and lower clustering and path length were observed, whereas in advanced disease stages elevated tau pathology was progressively associated with more brain network abnormalities. Moreover, the association between higher tau load and lower cognitive performance was only partly mediated (9.3 to 9.5%) through small world topology.

**Conclusions:**

Our data suggest a close relationship between grey matter network disruptions and tau pathology in individuals with abnormal amyloid. This might reflect a reduced communication between neighbouring brain areas and an altered ability to integrate information from distributed brain regions with tau pathology, indicative of a more random network topology across different AD stages.

**Supplementary Information:**

The online version contains supplementary material available at 10.1186/s13195-021-00876-7.

## Introduction

Alzheimer’s disease (AD) is generally thought to start with the aggregation of amyloid-β (Aβ) in the brain, followed by deposition of neocortical tau pathology, synaptic dysfunction, atrophy, and cognitive decline [1–3]. However, the sequence and interactions of the pathophysiological processes and structural brain changes that occur during this long pre-dementia period are not well understood. Given that brain network abnormalities can already be observed in pre-dementia stages and contribute to cognitive impairment in AD [4–6], further clarification on the interrelations between brain connectivity with key pathological aggregates of AD could increase our understanding on the pathogenesis of AD. One method to assess brain connectivity consists of measuring the similarity of cortical grey matter (GM) morphology, based on the notion that brain regions that engage in similar cognitive or behavioural processes tend to develop in a homologous way [7–9] through functional coactivation and/or axonal connectivity between brain regions [10–12]. Previous studies have shown GM network disruptions in preclinical AD [13–16], mild cognitive impairment [17–21], and AD dementia [5, 22–26]. Moreover, GM network disruptions have been related to increased Aβ pathology [13, 15, 16, 27], while influences of tau pathology on GM brain networks remain unknown. Intraneuronal tau is thought to be more closely linked to synaptic function, brain integrity and clinical symptoms, than Aβ plaques [28–31]. Therefore, we hypothesised that tau pathology may contribute to impaired network organisation in AD. In this study, we tested whether tau deposition (measured with tau-positron emission tomography [PET]) was associated with GM network alterations (measured with structural magnetic resonance imaging [MRI]) in individuals across the AD spectrum and whether these relationships were differentially linked with disease severity.

## Methods

### Participants

We included 533 individuals from the Swedish BioFINDER-2 study (NCT03174938) who underwent tau-PET, structural MRI, and lumbar puncture to determine cerebrospinal fluid (CSF) Aβ_42_/Aβ_40_ levels as described previously [32]. In the present study, we included subjects > 50 years of age with an abnormal CSF Aβ status, resulting in three groups along the AD continuum: Aβ-positive cognitively unimpaired (CU) subjects (preclinical AD), Aβ-positive patients with mild cognitive impairment (prodromal AD), and Aβ-positive patients with AD dementia. Diagnosis was made according to clinical diagnostic criteria of the diagnostic and statistical manual of mental disorders (DSM)-5 [33]. In addition, an Aβ-negative cognitively unimpaired control group was included. All subjects underwent the Mini-Mental State Examination (MMSE) [34] and delayed word list recall test from the ADAS-Cog (Alzheimer’s Disease Assessment Scale - Cognitive Subscale) [35] to assess global cognition and episodic memory, respectively. The inclusion and exclusion criteria of the BioFINDER-2 sub-cohorts are described in more detail in the eMethods section of the Supplementary material. All participants gave written informed consent to participate in the study. Ethical approval was given by the regional ethics committee at Lund University, Sweden. PET imaging procedures were approved by the Radiation protection committee at Skåne University Hospital and by the Swedish Medical Products Agency.

### MRI acquisition and pre-processing

T1-weighted images were acquired using a magnetisation-prepared rapid gradient echo sequence on a 3 T Siemens MAGNETOM Prisma scanner (Siemens Medical Solutions, Erlangen, Germany) using the following parameters: 178 slices, repetition time: 1950 ms, echo time: 3.4 ms, inversion time: 900 ms, flip angle: 9°, 1 mm isotropic voxels. All images were segmented into grey matter, white matter, and CSF using the Statistical Parametric Mapping (SPM12, https://www.fil.ion.ucl.ac.uk/spm/software/spm12/) running in MATLAB (v2019b). The segmented grey matter images were resliced to 2 × 2 × 2 mm isotropic voxels to reduce the dimensionality of the data. Then, the images were parcellated into 100 anatomical brain regions using the Automated Anatomical Labelling (AAL3) atlas (all thalamic nuclei combined) [36], which was warped from standard space to subject space using subject-specific inverse normalisation parameters. The quality of these steps was visually assessed and two subjects had to be excluded due to misalignment of the brain atlas with the GM image. Total intracranial volume (TIV) was computed as the sum of grey matter, white matter, and CSF volumes.

### Single-subject grey matter networks

Single-subject grey matter networks were extracted from the native grey matter images using an automated template-free approach that has been previously published (https://github.com/bettytijms/Single_Subject_Grey_Matter_Networks) [37]. For each subject, the grey matter network was built as a set of nodes connected by edges. The nodes were defined as cubes of 3 × 3 × 3 voxels (6 × 6 × 6 mm^3^), which size was chosen based on 2 factors: (I) the minimum spatial resolution needed to still capture cortical folding has shown to be 3 mm [38], and (II) the practical computational limitations that exist with large matrices. These cubes keep the 3D structure of the cortex intact, and thereby contain information on grey matter intensity as well as spatial information between the voxels. In order to find the maximum correlation value with a target cube using Pearson correlation coefficients, each cube was rotated by an angle with multiples of 45° over all axes, contributing to all positive connections. Self-connections were set to zero. The resulting similarity matrix containing all pairwise correlations was binarised using a threshold that reduced the chance of spurious correlations in the network to 5%. This corresponded to a significance level of *p* < 0.05 corrected for multiple comparisons using a permutation-based procedure [39]. The presence of an edge was indicated when the correlation between each pair of nodes exceeds this threshold. For regional network analyses, the corresponding atlas label for each cube was determined, this enabled averaging the GM network values and volume across nodes that were labelled according to that atlas region. A total of four subjects had to be excluded due to network calculation failure.

### Network properties

Global and regional measures were calculated for each individual GM network. To assess global network properties the following measures were computed: network size (the total number of nodes in the network), degree (the number of edges), connectivity density (the ratio of existing connections to the maximum number of possible connections), clustering coefficient (the fraction of a node’s neighbours that are also neighbours of each other), and path length (shortest path length between all pairs of nodes in the network). To normalise the network properties to random networks, we divided the average clustering coefficient and path length values by those values of five randomised reference networks of identical size and degree distribution, resulting in *γ* and *λ*, respectively [40]. The ratio of *γ* to *λ* is defined as the small-world coefficient σ [41], indicative of the optimal balance between information segregation and integration. To assess regional network properties, the degree, clustering coefficient, and path length were also calculated for each atlas brain area, i.e. region of interest (ROI). All network measures were computed with functions from the Brain Connectivity Toolbox (https://sites.google.com/site/bctnet/) [42], modified for large scale networks.

### PET acquisition and pre-processing

Tau-PET imaging was conducted 70–90 min after injection of 365 ± 20 MBq [^18^F]RO948 on digital GE Discovery MI scanners (General Electric Medical Systems) [43]. Low-dose CT scans were performed immediately prior to the PET scans for attenuation correction. PET data was reconstructed using VPFX-S (ordered subset expectation maximisation with time-of-flight and point spread function corrections) with 6 iterations and 17 subsets with 3 mm smoothing, standard Z filter, and 25.6-cm field of view with a 256 × 256 matrix. After list-mode data was binned into 4 × 5-min time frames, PET images were motion corrected (rigid transformation using AFNI, 3dvolreg) [44], summed, and co-registered to their corresponding T1-weighted MR images. Standardised uptake value ratio (SUVR) images were created using the inferior cerebellar cortex as a reference region [32]. We calculated PET data both corrected and uncorrected for partial volume errors. Partial volume correction (PVC) was performed using the geometric transfer matrix method, as described in [45], PVC findings are available in the supplementary results section (eFigure 1, 2, eTable2).

To investigate the associations between tau pathology and network changes across different AD pathological stages, four composite regions were created based on the Braak staging scheme for neurofibrillary tangle pathology [46], adapted to PET space by [47]. These included the following brain regions as defined by the AAL atlas (see Supplementary eMethods 2 for details), and cover stage I–II (hippocampal formation), stage III–IV (fusiform, amygdala, cingulate and inferior and middle temporal cortices), and stage V–VI (widespread neocortical areas including the orbitofrontal, superior, middle and inferior frontal, precentral, paracentral, postcentral, precuneus, inferior and superior parietal, supramarginal, superior temporal, medial and lateral occipital cortices). In addition, a tau temporal meta-ROI capturing stages I to IV was calculated using the volume-weighted average of the corresponding regions [48].

### CSF collection and analysis

CSF samples were obtained with a lumbar puncture and collected into 5 ml LoBind polypropylene tubes handled according to the Alzheimer’s Association Flow Chart for lumbar puncture [49]. Concentrations of Aβ_42_ and Aβ_40_ were quantified using enzyme-linked immunosorbent assays (ELISAs; INNOTEST, Fujirebio). Amyloid-status was determined using the Aβ_42_/Aβ_40_ ratio with a cutoff of < 0.089 as defined abnormal in clinical practice at the Sahlgrenska University Hospital, Mölndal, Sweden [32].

### Statistical analyses

Comparisons of demographical and clinical characteristics between groups were performed using chi-squared tests for categorical variables and one-way ANOVA for continuous variables.

To analyse the relationship between tau-PET (predictor) and global network measures (outcome), we used linear regression models adjusted for age, sex, and TIV (all models) and connectivity density (for path length, clustering, *γ*, *λ*, and *σ*), since higher-order measures have shown to depend on the number of nodes and edges in the network [50]. The association between tau-PET and global network measures was first tested across the whole sample, and then repeated within diagnostic groups with the interaction term group. Additionally, CSF Aβ_42_/Aβ_40_ was added to the linear regression model to test for an interaction effect CSF Aβ_42_/Aβ_40_* tau on GM network changes. *Z*-scores for the network properties were calculated using the Aβ-negative control group mean and standard deviation, to aid comparisons of effect sizes between network properties. We further investigated the relationship of tau pathology with GM network disruptions at the local level across all AAL areas. Regional analyses were adjusted for age, sex, TIV, local GM volume, and for clustering and path length also local degree. For global network analyses, we applied a threshold of *p* < 0.05, corrected for multiple comparisons using the false discovery rate (FDR) correction method. For local network analyses, we adjusted for multiple testing using a Bonferroni correction (*p* < 0.05). Finally, we performed a mediation analysis using the Mediation package [51], to assess whether the association of tau pathology with cognitive performance was mediated by GM network disruptions. For these exploratory analyses, we assessed the small world coefficient only, as it indicates how randomly organised the network is, and it can be considered a summary measure of both *γ* and *λ*. Analyses were performed using R (v4.0.2.) and visualised using Surf Ice (v2).

## Results

### Participants

In total, 178 Aβ-negative CU controls, 105 preclinical AD, 122 prodromal AD, and 128 AD dementia patients were included in the present study (mean age = 70.5 ± 9.3 years; Table 1). Control subjects were younger and had a lower prevalence of apolipoprotein E (*APOE*) *ε4* than the other groups. As expected, lower MMSE scores, lower hippocampal volume, and higher tau-PET SUVr values were observed in the prodromal AD and AD dementia groups compared to the CU subjects.

**Table 1:**
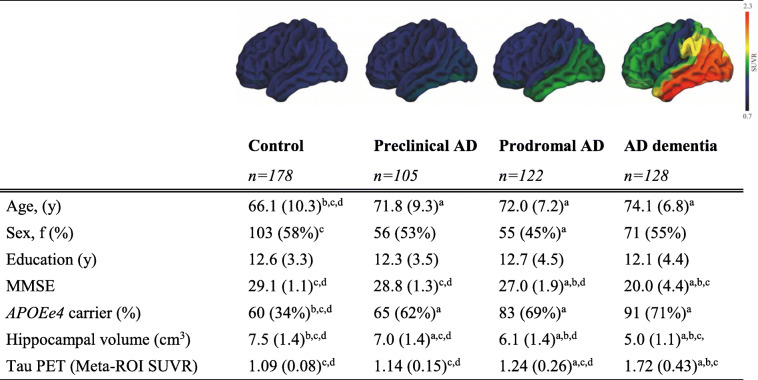
Subject characteristics

Data are presented as mean ± SD, or n (%); AD, Alzheimer's disease; F, female; MMSE, Mini Mental State Examination (0–30); ApoE, Apolipoprotein E; SUVR, Standardized uptake value ratio; ApoE 2 missing’s; *a* significantly different than control; *b* significantly different than preclinical AD; *c* significantly different than prodromal AD; *d* significantly different than AD dementia at *p*<0.05; Surface plots display mean [18F]RO948 SUVR images.

### Single-subject grey matter networks

Networks had an average size of 6976 nodes (sd=664) and an average connectivity density of 16% (sd=1) across all participants (Table 2). We observed that all grey matter network metrics showed lower values indicative of increased network abnormalities with advancing disease severity (Table 2). Differences between Aβ-negative controls and preclinical AD subjects were subtle and higher-order network differences lost significance after adjusting for covariates, including age. Compared to Aβ-negative controls, prodromal AD subjects showed lower clustering, path length, *γ*, *λ*, and small world coefficient values. Similar changes were observed in AD dementia, with in addition lower network size and degree compared to controls. In addition, all higher-order metrics were significantly lower in AD dementia compared to prodromal AD and significantly lower in prodromal AD compared to preclinical AD.

**Table 2:** Summary of global network measures

	Control *n* = 178	Preclinical AD *n* = 105	Prodromal AD *n* = 122	AD dementia *n* =128
Network size	7171.15 (671.92)^d^	7007.46 (672.18)^d^	6987.52 (612.17)^d^	6666.01 (581.66)^a, b, c^
Degree	1193.11 (148.21) ^d^	1159.10 (139.57)^d^	1140.61 (133.61)^d^	1072.01 (138.78)^a, b, c^
Connectivity density	16.61 (0.98)	16.53 (1.07)	16.30 (0.92)	16.06 (1.27)
Clustering coefficient	0.46 (0.02)^c, d^	0.46 (0.02)^c, d^	0.45 (0.02)^a, b, d^	0.44 (0.02)^a, b, c^
Path length	1.98 (0.03)^c, d^	1.97 (0.03)^c, d^	1.96 (0.03)^a, b, d^	1.96 (0.03)^a, b, c^
Gamma	1.58 (0.11)^c, d^	1.54 (0.12)^c, d^	1.50 (0.09)^a, b, d^	1.43 (0.09)^a, b, c^
Lambda	1.08 (0.01)^c, d^	1.08 (0.02)^c, d^	1.07 (0.01)^a, b, d^	1.06 (0.01)^a, b, c^
Small world coefficient	1.47 (0.08)^c, d^	1.43 (0.09)^c, d^	1.40 (0.07)^a, b, d^	1.35 (0.07)^a, b, c^

Data are presented as mean ± SD; *AD* Alzheimer’s disease; gamma is normalised clustering; lambda is normalised path length; ^a^significantly different than control; ^b^significantly different than preclinical AD; ^c^significantly different than prodromal AD; ^d^significantly different than AD dementia at *p* < 0.05 when adjusting for age, sex, TIV, and connectivity density (clustering, path length, *γ*, *λ,* small world-only)

### Relationship between tau pathology and global grey matter network measures

**Fig. 1:**
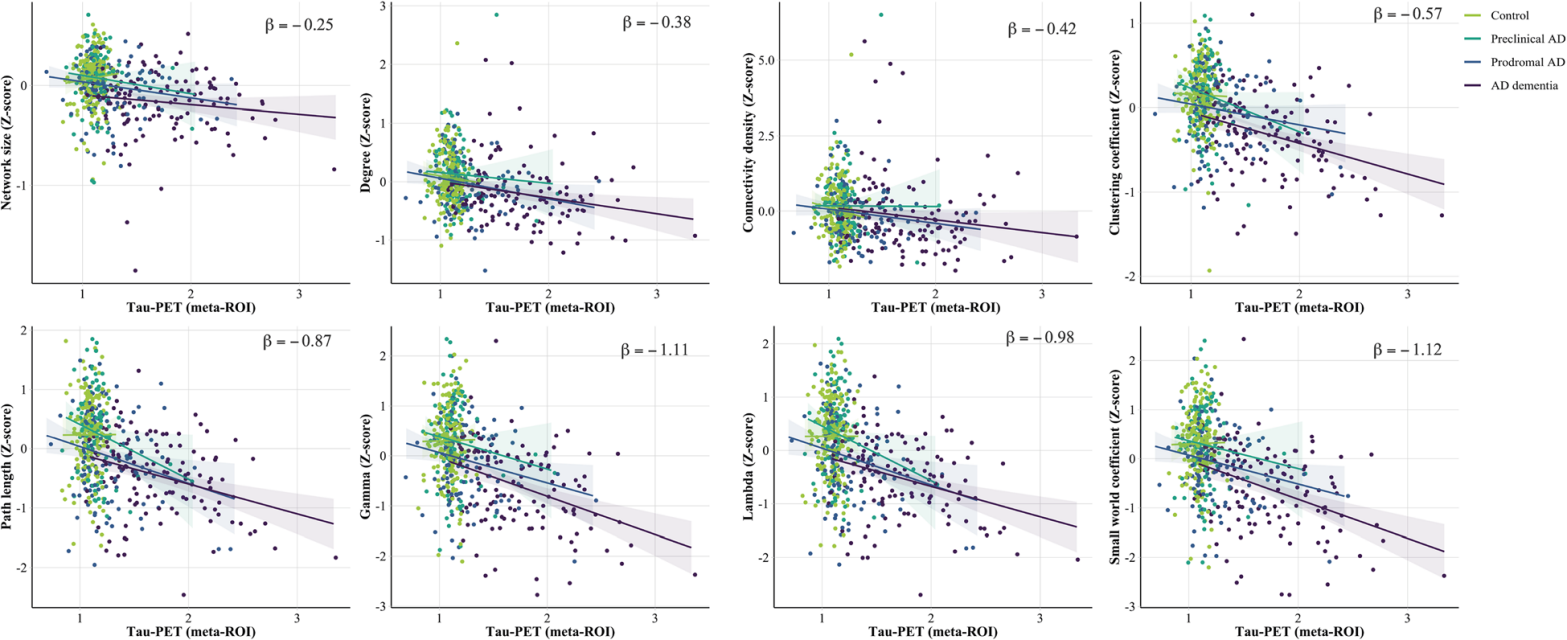
Scatterplots of the relation between tau-PET SUVR values and global grey matter network measures by disease stage. Standardised beta estimates are displayed for significant relationships across all participants adjusting for age, sex, TIV, and connectivity density (clustering, path length, *γ*, *λ*, small world-only)

Across the whole sample, higher tau-PET values in the temporal meta-ROI were associated with lower values in all network properties (range *β* = − 0.25 to *β* = − 1.12; Fig. 1; Table 3), showing strongest associations for γ and small world values. We observed similar relationships between GM network measures and tau signal in early (i.e. stage I/II) and late (i.e. stage V/VI) tau accumulation regions (Supplementary Results eTable 1).

**Table 3:** Associations between global network measures and tau-PET

Network property	Whole sample	Control	Preclinical AD	Prodromal AD	AD dementia
Network size	− 0.25 (0.03)^***^	0.14 (0.26)	− 0.22 (0.18)	− 0.17 (0.10)	− 0.08 (0.06)
Degree	− 0.38 (0.06)^***^	− 0.37 (0.46)	− 0.22 (0.31)	− 0.36 (0.17)	− 0.26 (0.10)^*^
Connectivity density	− 0.42 (0.13)^**^	− 1.03 (0.97)	− 0.04 (0.66)	− 0.48 (0.35)	− 0.42 (0.21)
Clustering coefficient	− 0.57 (0.05)^***^	− 0.24 (0.38)	− 0.58 (0.26)^*^	− 0.26 (0.14)	− 0.34 (0.08)^***^
Path length	− 0.87 (0.08)^***^	− 0.25 (0.62)	− 1.04 (0.42)^*^	− 0.65 (0.23)^**^	− 0.47 (0.14)^**^
Gamma	− 1.11 (0.09)^***^	− 0.23 (0.71)	− 0.79 (0.48)	− 0.65 (0.25)^*^	− 0.72 (0.16)^***^
Lambda	− 0.98 (0.09)^***^	− 0.29 (0.69)	− 1.15 (0.47)^*^	− 0.72 (0.25)^**^	− 0.53 (0.15)^**^
Small world coefficient	− 0.12 (0.10)^***^	− 0.21 (0.73)	− 0.70 (0.49)	− 0.63 (0.27)^*^	− 0.75 (0.16)^***^

Data are presented as β (SE); Network measures are Z trasformed; gamma is normalised clustering; lambda is normalised path length; SUVr values in temporal meta-ROI; Model is adjusted for age, sex, TIV, and connectivity density (clustering, path length, *γ*, *λ,* small world-only). *p < 0.05, **p <0.01, ***p < 0.001, FDR corrected.

When repeating the analyses stratified by diagnostic group, we found more significant associations between tau-PET and network measures with increasing disease severity: In preclinical AD, higher tau-PET values in the meta-ROI were associated with lower clustering values (*β*±SE; − 0.58±0.26), path length (− 1.04±0.24), and *λ* values (− 1.15±0.47; all *p* < .05; Fig. 1; Table 3). In prodromal AD, higher tau-PET retention was related to lower path length (− 0.65±0.23), *γ* (− 0.65±0.26), *λ* (− 0.72±0.25), and small world coefficient values (− 0.63±0.27). In AD dementia, higher tau PET signal in the temporal meta-ROI was associated with lower degree (− 0.26±0.10), clustering (− 0.34±0.08), path length (− 0.47±0.14), γ (− 0.72±0.16), *λ* (− 0.53±0.15), and small world coefficient values (− 0.75±0.16). No association between tau-PET values and global network measures was observed in Aβ-negative controls. Moreover, no group interaction effects were observed, indicating that the *strength* of the association between GM network measures and tau pathology did not differ significantly between disease stages.

When repeating the same analysis using the three ROIs specific for different tau stages, no association was observed between tau-PET values in the hippocampal formation (Braak I/II) and global network properties in preclinical AD participants (Supplementary Results eTable 1), suggesting that using the tau-PET signal in the temporal meta-ROI is more suitable in relation to network measures than early tau-accumulation regions. Tau pathology in limbic (Braak III-IV) and neocortical (Braak V-VI) areas correlated with decreased degree, clustering, path length, *γ*, *λ*, and small world coefficient values in prodromal and AD dementia subjects (Supplementary Results eTable 1). Moreover, we observed similar relationships between GM network measures and tau signal when repeating the analyses without the Aβ-negative control group (Supplementary Results eTable 3). Furthermore, there were no significant interactions between tau pathology and Aβ_42_/Aβ_40_ ratio values on GM network measures, suggesting that the associations between GM network alterations and tau pathology in Aβ positive individuals cannot be explained by CSF Aβ levels (Supplementary Results eTable 3).

### Relationship between tau pathology and regional grey matter network measures

**Fig. 2:**
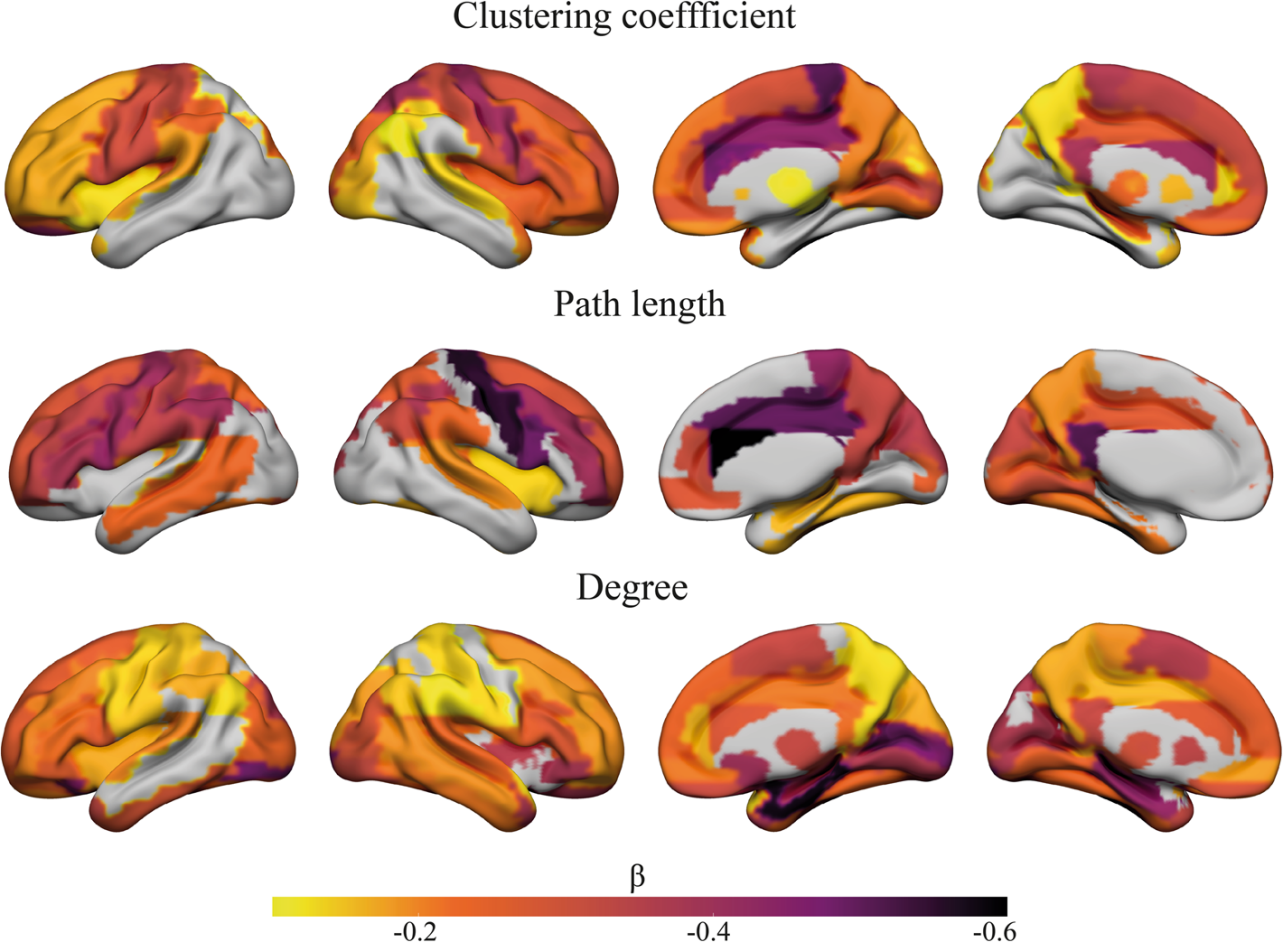
Surface plots of standardised β values of the relationship between tau-PET and local clustering, path length, and degree in participants with abnormal Aβ. Data are presented for regions with a significant correlation at *p*_Bonferroni_ < .05 adjusted for age, sex, TIV, local degree, and local GM volume

Next, we examined the relationship between tau burden in the temporal meta-ROI with network measures at a local level in subjects on the AD continuum to assess a region-dependent effect. We observed different anatomical patterns of associations for each network measure. Lower clustering values were associated with higher tau-PET SUVr values in widespread brain areas, showing the strongest associations in the precentral cortex, cingulate gyri, and frontal lobe (all *p*_bonferroni_ < 0.05; Fig. 2). Associations between lower path length values and higher tau-PET retention were strongest in the cingulate gyri, precentral cortex, and inferior frontal cortex. Regions characterised by a lower degree showed also widespread associations with tau, which was most pronounced in the hippocampus, parahippocampus, amygdala, medial occipital cortex, and calcarine cortex (Fig. 2).

### Associations with cognitive performance

**Fig. 3:**
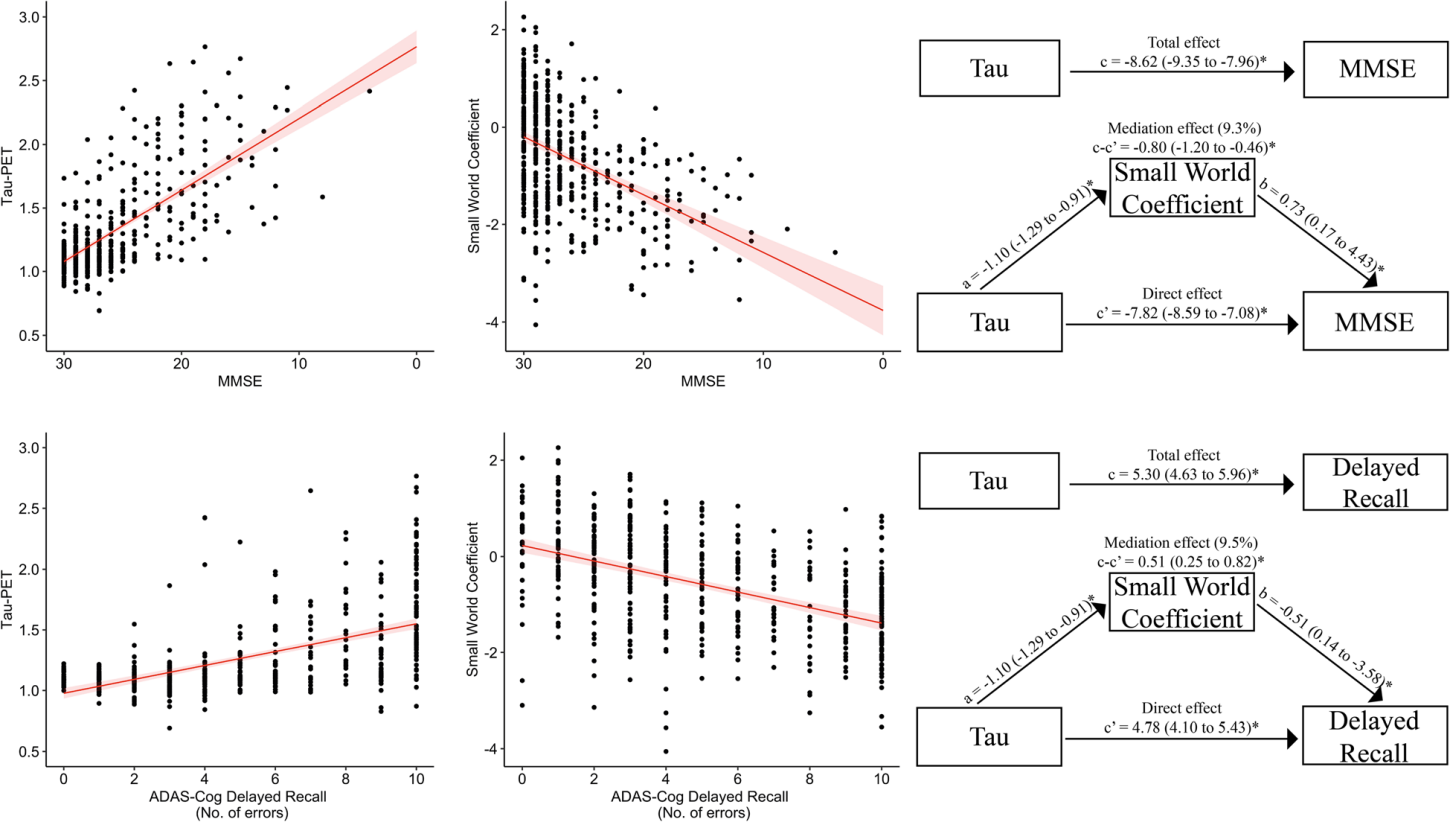
MMSE and ADAS-Cog delayed recall performance in relation to Tau-PET and Small world coefficient. Scatterplot showing the association of tau-PET SUVR values in the temporal meta-ROI and standardised grey matter network small world values with MMSE scores (top), and the ADAS-Cog delayed recall (bottom). Mediation analysis showing how small world topology mediates the effect of tau pathology on cognitive performance (right). Regression coefficients with a 95% confidence interval are displayed. **a** The effect of tau load on small world topology. **b** The effect of small world topology on cognitive performance when controlling for tau. **c** The total effect of tau on cognition (without controlling for mediation effects). **c’** The direct effect of tau pathology on cognition when adjusting for mediation. **c**–**c’**, The mediation effect. *p < 0.05

We performed an exploratory analysis of the relationship of tau-pathology and small world topology with cognitive performance. Both higher tau-PET retention (*β* ± SE; − 8.6±0.37; 5.30±0.34) and lower small world values (*β* ± SE; 2.14±0.19; − 1.23±0.15) were significantly related to lower MMSE scores and more errors on the ADAS-Cog delayed word recall, respectively (*p*< 0.05; Fig. 3). Moreover, small world values remained significant when controlling for tau-SUVr values. Mediation analysis revealed that lower MMSE scores and more recall errors associated with tau pathology were partially mediated by decreased small world values (9.3.0 to 9.5%), but were mostly independent after controlling for the effect of small world coefficient.

## Discussion

In a large well-phenotyped cohort, we observed that higher tau-PET retention is related to greater GM network disruptions in individuals across the AD continuum. More advanced tau-related GM network abnormalities were observed with increasing disease severity. These findings suggest that tau pathology is associated with a reduced communication between neighbouring brain areas and an altered ability to integrate information from distributed brain regions indicative of a more random network topology across different AD stages.

Our results show a close relationship between GM network disruptions and tau pathology in individuals with abnormal amyloid. With increasing disease severity, we observed a greater tau load, as well as a greater number of abnormal GM network measures. As a result, the association of more network abnormalities with increased tau-PET retention may be largely based on differences in disease stage. Furthermore, when stratifying the analyses for diagnostic groups, we observed that distinct network measures were sensitive to different aspects and severity of neurodegeneration. Specifically, already in preclinical AD increased tau-PET signal was associated with network alterations showing lower clustering, lower path length values and normalised path length *λ*. These associations were absent in CU individuals with normal amyloid, indicating that the presence of Aβ might significantly alter tau-related GM connectivity. These findings are in line with prior studies showing deterioration in network properties in preclinical AD [13, 14, 52]. In prodromal AD more network measures became abnormal with increasing tau pathology, additionally including changes in normalised clustering *γ*, and small world topology. In AD dementia patients nearly all network metrics were abnormal, suggesting that with pronounced neurodegeneration GM networks exhibit significant topological alterations, which is in line with previous studies in AD dementia [18, 22, 23]. Overall, these findings may indicate that a higher tau burden is associated with a higher dissimilarity (i.e. asynchronous atrophy) between neighbouring areas, and increased similarity between distant brain areas as a result of the progression of atrophy across the cortex, producing an increase in randomly connected nodes. Suggestive of an increasingly random network and reduction in small world organisation with disease progression [4, 42].

When characterising the spatial associations of tau pathology and GM network changes, tau deposition appeared to be widespread and strongly associated with lower clustering and path length in several regions of the default-mode network including the medial prefrontal cortex, precuneus, and anterior and posterior cingulate cortex. These findings may be related to early amyloid accumulating regions [53, 54]. We see that it is in these regions that clustering and path length further relates to tau, while also, more unexpectedly, in late tau accumulating regions such as the sensory-motor cortex and occipital lobe, strong associations of lower clustering and path length with tau were observed. Similar regional associations of GM network decline over time have been observed in individuals in presymptomatic stages of familial AD [55]. For lower degree, most strong correlations were observed in the medial temporal lobe, a region considered to be the tau epicentre where neurofibrillary tangles originate [46], suggestive of less connections in the medial temporal lobe with increasing tau-PET signal.

Previous studies have related alterations in GM networks with abnormal amyloid aggregation in cognitively unimpaired individuals [13, 15, 16, 27], indicating that structural changes in GM networks are an early event in the pathophysiology of AD. Moreover, the presence of Aβ is hypothesised to increase the accumulation of tau outside of the medial temporal lobe [56–58], which may further accelerate network decline. As pathological tau shows reduced ability to stabilise microtubules, contributing to impaired axonal transport [30, 31], this may lead to further synaptic loss and neurodegeneration, resulting in substantial network damage and impaired cognition [28, 29, 59, 60]. As reflected by our work showing that both increased tau pathology and a more random GM network topology were associated with worse performance on a global cognition and episodic memory test. Both factors showed an independent contribution to worse cognitive performance, while mediation analyses also indicated that small world topology party mediated the effect of tau pathology on cognition.

## Limitations

There are some limitations to our study. Firstly, this is a cross-sectional study that assumes three clinical stages of disease progression, and future longitudinal studies are needed to determine the temporal ordering in tau pathology and associated brain network changes more accurately. Secondly, for uniformity reasons, ROIs for both regional tau quantification and network topology calculation were created according to the AAL atlas. Unfortunately, the entorhinal cortex is not available as a separate region in this brain atlas; hence, in the current study stage I/II refers to the hippocampus and parahippocampus which includes the entorhinal cortex. This may have attenuated some of the results, but when testing the accuracy of the temporal meta-ROI in the AAL atlas with the Desikan-Killiany atlas, we observed a correlation of *R* = .99, rendering such effects likely to be minimal. Thirdly, since tau abnormalities are closely related to Aβ pathology, it is difficult to know how specific the observed GM network alterations are to tau pathology. Strengths of our study include a large number of well-characterised participants. Moreover, the multimodal approach of combining structural MRI and tau-PET imaging aids in understanding fundamental questions in the AD pathophysiology.

## Conclusions

We found that GM network disruptions in AD are strongly linked with tau burden, already in an early disease stage when cognition is within the normal range and becomes increasingly random with clinical progression. These findings provide more insight into the pathophysiological processes that contribute to brain network alterations in AD. An interesting future approach might lie in further investigating the prognostic value of GM single-subject networks in predicting cognitive decline and whether it can be implemented in clinical practice.

## Supplementary Information

**Additional file 1. **Methods e1. Inclusion and exclusion criteria for the Swedish BioFINDER 2 study. Methods e2. AAL atlas ROIs included in Braak staging. Results eTable 1. Results eTable 2. Results eTable 3. Results eFigure 1. Scatterplots of the relation between tau SUVr with PVC and global grey matter network measures by disease stage. Standardised beta estimates are displayed for significant relationships across all participants adjusting for age, sex, TIV, and connectivity density. Results eFigure 2. Surface plots of standardised β values of the relationship between tau SUVr with PVC and local clustering, path length, and degree in participants with abnormal Aβ. Data are presented for regions with a significant correlation at *p*_Bonferroni_<.05 adjusted for age, sex, TIV, local degree, and local GM volume.

## Data Availability

Anonymised study data for the primary analyses presented in this report are available on request from any qualified investigator for purposes of replicating the results.

## Abbreviations

AAL: Automated Anatomical Labelling
AD: Alzheimer’s disease
Aβ: Amyloid-β
APOE: Apolipoprotein E
CSF: Cerebrospinal fluid
CU: Cognitively unimpaired
DSM: Diagnostic and Statistical Manual of Mental Disorders
FDR: False discovery rate
GM: Grey matter
MMSE: Mini-Mental State Examination
MRI: Magnetic resonance imaging
PET: Positron emission tomography
PVC: Partial volume correction
ROI: Region of interest
SPM: Statistical Parametric Mapping
SUVR: Standardised uptake value ratio
TIV: Total intracranial volume
