# Supplementary material for: Tau-related grey matter network breakdown across the Alzheimer’s disease continuum
Source: Alzheimers Res Ther. 2021 Aug 13;13:138. doi: 10.1186/s13195-021-00876-7 (PMC8364121; doi:10.1186/s13195-021-00876-7)
Supplement: Supplementary file 1 — Additional file 1. Methods e1. Inclusion and exclusion criteria for the Swedish BioFINDER 2 study. Methods e2. AAL atlas ROIs included in Braak staging. Results eTable 1. Results eTable 2. Results eTable 3. Results eFigure 1. Scatterplots of the relation between tau SUVr with PVC and global grey matter network measures by disease stage. Standardised beta estimates are displayed for significant relationships across all participants adjusting for age, sex, TIV, and connectivity density. Results eFigure 2. Surface plots of standardised β values of the relationship between tau SUVr with PVC and local clustering, path length, and degree in participants with abnormal Aβ. Data are presented for regions with a significant correlation at pBonferroni<.05 adjusted for age, sex, TIV, local degree, and local GM volume. [file 13195_2021_876_MOESM1_ESM.docx]

﻿**Supplementary material**

**Methods e1. Inclusion and exclusion criteria for the Swedish BioFINDER 2 study**

The BioFINDER-2 study enrols participants in five sub-cohorts; Cohort A and B includes neurologically and cognitively healthy controls. The inclusion criteria are: i) ages 40-65 years (cohort A) and ages 66-100 years (cohort B); ii) absence of cognitive symptoms as assessed by a physician with special interest in cognitive disorders; iii) MMSE score 27-30 (A) or 26-30 (cohort B) at screening visit; iv) do not fulfil the criteria for MCI or any dementia according to DSM-5 [1]; v) fluent in Swedish. The recruitment process of cohorts A and B is designed to build two study populations with 50% *APOE* ε4 carriers in each.

Cohort C comprises participants with subjective cognitive deficits (SCD) or minor neurocognitive impairment (MCI) (the latter according to DSM-5 [1]. Inclusion criteria are: i) Age 40-100 years; ii) referred to the memory clinics due to cognitive symptoms; iii) MMSE score of 24 – 30 points; iv) does not fulfil the criteria for any dementia (major neurocognitive disorder) according to DSM-5 [1], v) fluent in Swedish. In accordance with the research framework by the National Institute on Aging-Alzheimer’s Association [2] study participants with SCD were analysed together with the cognitively healthy participants (and combined in the cognitively unimpaired group). Participants were classified as having MCI if they performed worse than -1.5 SD in any cognitive domain according to age and education stratified test norms. The neuropsychological battery covered the domains attention/executive function (Trail Making Test A and B, Symbol Digit Modalities Test, and AQT), memory (10 word immediate and delayed recall from the Alzheimer’s Disease Assessment Scale [ADAS]), verbal ability (verbal fluency and the short version of the Boston Naming Test) and visuospatial function (incomplete letters and cube analysis from the Visual Object and Space Perception battery). Those that were not classified as MCI were considered to have SCD.

Cohort D consists of participants with dementia due to AD. Inclusion criteria are: i) Age 40-100 years; ii) referred to the memory clinics due to cognitive symptoms; iii) MMSE score of ≥12 points; iv) fulfil the DSM-5 criteria for dementia (major neurocognitive disorder) due to Alzheimer’s disease [1]; v) fluent in Swedish. Cohort E covers other non-AD dementias and neurodegenerative disorders. Inclusion criteria are: i) Age 40-100 years; ii) fulfilment of criteria for dementia (major neurocognitive disorder) due to frontotemporal dementia [1], Parkinson’s disease with dementia, dementia with Lewy Bodies or vascular dementia [1] alternatively the criteria for Parkinson’s disease [3], progressive supranuclear palsy [4], multiple system atrophy [5], or semantic variant primary progressive aphasia [6]; iii) fluent in Swedish.

Exclusion criteria for all sub-cohorts are: i) significant unstable systemic illness that makes it difficult to participate in the study; ii) current significant alcohol or substance misuse; iii) refusing lumbar puncture, MRI or PET.

**Methods e2. AAL atlas ROIs included in Braak staging**

**Results eTable 1.**

**Results eTable 2.**

**Results eTable 3.**

**Results eFigure 1.**

**
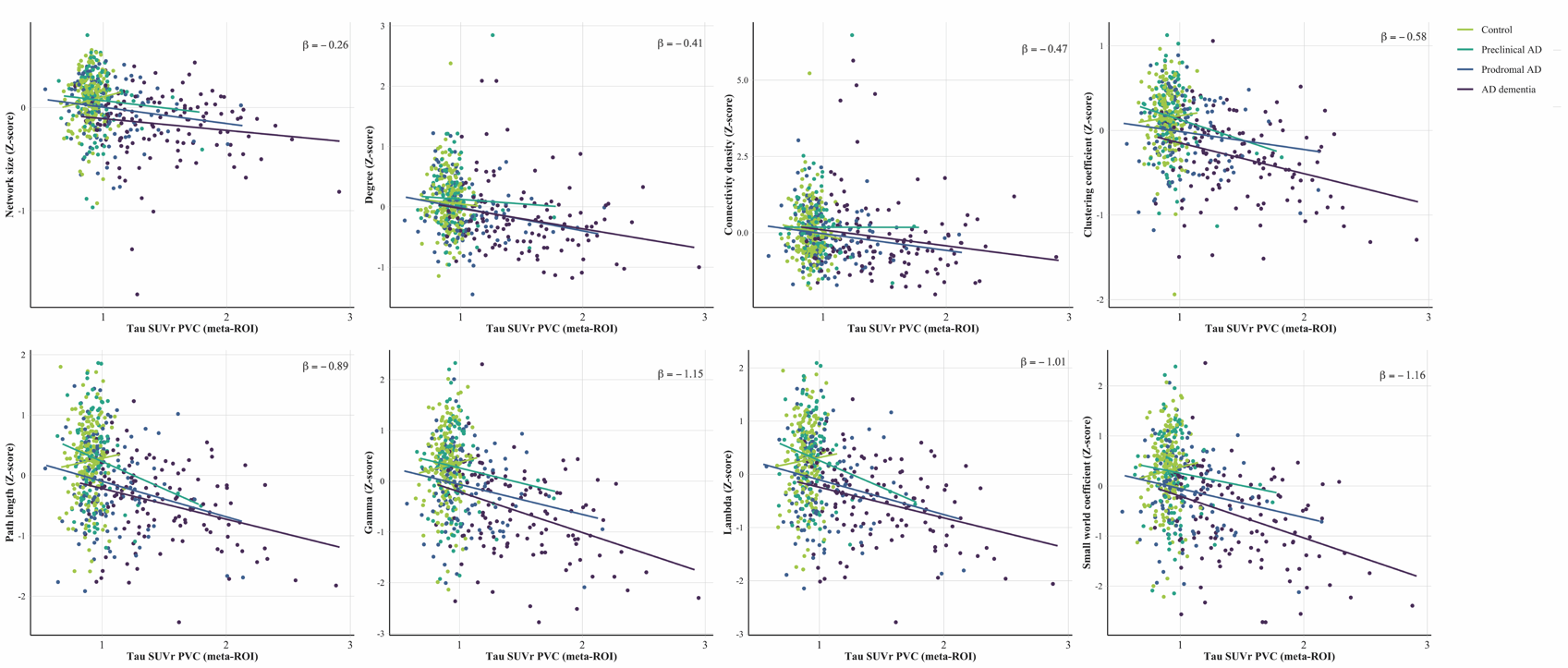
**

**Results eFigure 1**. Scatterplots of the relation between tau SUVr with PVC and global grey matter network measures by disease stage. Standardised beta estimates are displayed for significant relationships across all participants adjusting for age, sex, TIV, and connectivity density.

**Results eFigure 2.**

**
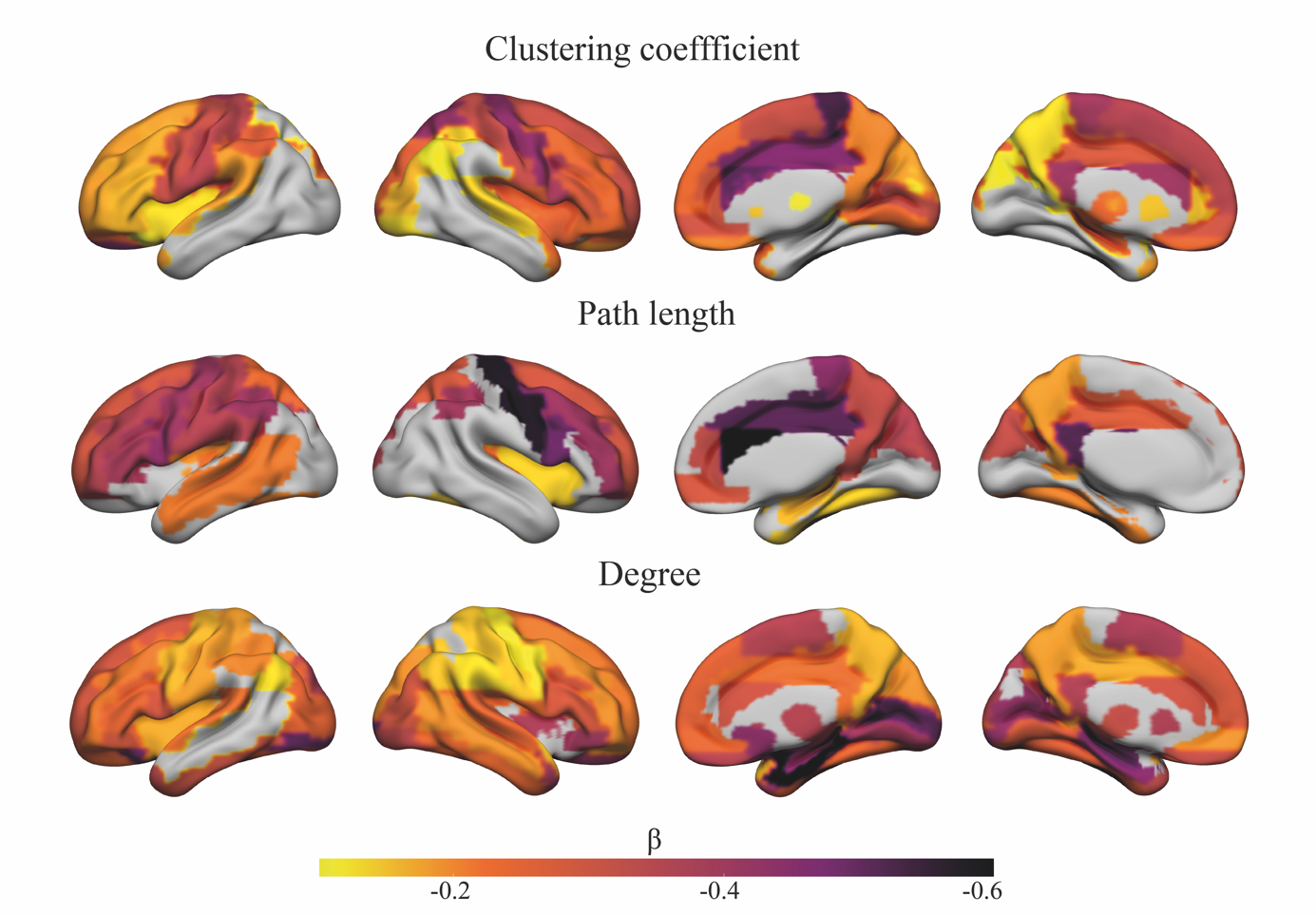
**

**Results eFigure 2.** Surface plots of standardised β values of the relationship between tau SUVr with PVC and local clustering, path length, and degree in participants with abnormal Aβ. Data are presented for regions with a significant correlation at *p*_Bonferroni_<.05 adjusted for age, sex, TIV, local degree, and local GM volume.

**References**

1. American Psychiatric Association (2013) Diagnostic and Statistical Manual of Mental Disorders. Arlington. https://doi.org/10.1176/appi.books.9780890425596

2. Jack CR, Bennett DA, Blennow K, et al (2018) NIA-AA Research Framework: Toward a biological definition of Alzheimer’s disease. Alzheimer’s and Dementia 14:535–562

3. Gelb DJ, Oliver E, Gilman S (1999) Diagnostic Criteria for Parkinson Disease. Archives of Neurology 56:33

4. Höglinger GU, Respondek G, Stamelou M, et al (2017) Clinical diagnosis of progressive supranuclear palsy: The movement disorder society criteria. Movement Disorders 32:853–864

5. Gilman S, Wenning GK, Low PA, et al (2008) Second consensus statement on the diagnosis of multiple system atrophy. Neurology 71:670–676

6. Gorno-Tempini ML, Hillis AE, Weintraub S, et al (2011) Classification of primary progressive aphasia and its variants. Neurology 76:1006–1014
